# Heavy menstrual bleeding and dysmenorrhea are improved by Magnetic Resonance Guided Focused Ultrasound Surgery (MRgFUS) of adenomyosis

**DOI:** 10.1186/s40738-016-0021-x

**Published:** 2016-05-16

**Authors:** Ramya Jayaram, Kalpana Subbarayan, Sridurga Mithraprabhu, Mirudhubashini Govindarajan

**Affiliations:** 1 Department of Obstetrics and Gynecology, Womens Center, 146B Mettupalayam Road, Coimbatore, 641043 Tamil Nadu India; 2 Department of Radiology, Womens Center, 146B Mettupalayam Road, Coimbatore, 641043 Tamil Nadu India; 3 Australian Centre for Blood Diseases, Alfred Hospital/Monash University, Melbourne, Victoria 3800 Australia

**Keywords:** MRI guided Focussed Ultrasound Surgery (MRgFUS), Adenomyosis, Uterus sparing surgery, Fertility preservation

## Abstract

**Background:**

To assess reduction in heavy menstrual bleeding and dysmenorrhea following MRI guided Focused Ultrasound Surgery (MRgFUS) of focal and diffuse adenomyosis up to 12 months post-treatment a retrospective cohort study was done at a tertiary care academic medical center for obstetrics, gynecology and infertility.

**Methods:**

MRgFUS for adenomyosis uterus was done for thirty-seven patients presenting with symptoms of heavy menstrual bleeding and dysmenorrhea with MRI-suspected adenomyosis. The main outcome measure, was reduction in heavy menstrual bleeding, dysmenorrhea and Symptom Severity Scoring (SSS) over a 3, 6 and 12 month period. Secondary outcome was evidence of fertility preservation post procedure. D’Agostino & Pearson omnibus normality test, one-way Ananova, Pearson’s correlation coefficient analysis was performed on the data. Statistical significances, p-value and r-value were determined.

**Results:**

Out of 37 patients who were treated by MRgFUS, 26 had sufficient follow-up to be included in the analysis. SSS calculated at 3, 6 and 12 months was significantly over the baseline. Both heavy menstrual bleeding and dysmenorrhea, which were assessed separately, were found to significantly improve over time with a positive correlation between the two. No other intervention was required.

**Conclusion:**

MRgFUS provides immediate and sustained relief for patients with focal and diffuse adenomyosis.

**Electronic supplementary material:**

The online version of this article (doi:10.1186/s40738-016-0021-x) contains supplementary material, which is available to authorized users.

## Background

Adenomyosis is defined as a benign invasion of endometrium into the myometrium, producing a diffusely enlarged uterus which microscopically exhibits ectopic, non-neoplastic, endometrial glands and stroma surrounded by the hypertrophic and hyperplastic myometrium [1]. While adenomyosis is strictly a pathological diagnosis, there is consensus now that MRI indicates the presence of adenomyosis when the junctional zone (JZ) thickness is > 12 mm [2].

Adenomyosis is usually diagnosed between 30 and 45 years of age [3], with an 8–27 % prevalence [4]. The increased incidence of younger women presenting with infertility and subsequently being diagnosed with adenomyosis may be the result of enhanced diagnostic capabilities. MRI is currently regarded as the best imaging tool for differential diagnosis between uterine fibroids and adenomyosis [5, 6]. However, MRI is not routinely used for the evaluation of these pathologies. A correct differential diagnosis is critical, because the optimal treatment modalities for these two entities may differ. Specifically, in women with symptomatic leiomyomas, uterine-conserving surgical therapy is well-established, whereas hysterectomy remains the treatment of choice for debilitating adenomyosis [7]. In contrast to uterine leiomyoma, adenomyosis is not demarcated from the adjacent myometrium by a capsule. Difficulty in defining the location and the extent of adenomyosis often makes it difficult to determine the feasibility and extent of complete excision when attempting to preserve the uterus [8]. Women with diffuse disease are often not considered as candidates for conservative surgery. Removal of adjacent healthy myometrium may increase the risk of bleeding and extensive damage to the uterine wall and could negatively affect the tensile strength of the uterus during pregnancy and labour and, thus, the ability of the uterus to carry future pregnancies safely to term [8, 9].

Symptoms of adenomyosis include heavy menstrual bleeding, dysmenorrhea and pelvic pressure and frequent urination from uterine enlargement. The severity of symptoms correlates roughly with the extent of disease [2, 10, 11]. Newer reports using MRI criteria for diagnosis suggest that the disease may cause dysmenorrhea and chronic pelvic pain in adolescents and women of younger reproductive age than previously appreciated [12, 13].

Magnetic resonance-guided focused ultrasound surgery (MRgFUS) has been used successfully over the past years for the conservative treatment of uterine leiomyomas, without damage to surrounding healthy myometrium and without major morbidity [9, 14]. It is a non-invasive technique for the ablation of soft tissue that offers a new approach to conservative non-invasive uterine surgery [9] Sequential ultrasound beams are precisely focused on a series of small foci within the larger target volume to locally heat tissue under real-time MR guidance and control, causing thermal coagulation and subsequent tissue necrosis in a precisely defined area. The first published case report of a pregnancy and live birth after focused ultrasound surgery for symptomatic focal adenomyosis was by Rabinovici et al [15]. Owing to the accuracy and precision of this technique, it has been hypothesized that MRgFUS could also treat adenomyosis accurately and successfully without deleterious effects on the surrounding myometrium and on subsequent fertility [15].

Previously published literature on the utilisation of MRgFUS for adenomyosis with short-term (3 and 6 months) and long-term (upto 2 years) follow-up has indicated that the treatment was safe and that there was significant reduction in symptoms overall [11, 16, 17]. However, the Symptom Severity Score (SSS) (UF-QOL) scoring which is used to assess the symptoms of patients before and after MRgFUS treatment for fibroids, is not specific for adenomyosis and does not assess dysmenorrhea, one of the most debilitating symptoms associated with adenomyosis.

The objective of this study was to evaluate the degree of reduction of dysmenorrhea and heavy menstrual bleeding after treatment with MRgFUS in patients with focal and diffuse adenomyosis and to determine whether MRgFUS, as a primary treatment modality, can provide both immediate and sustained symptom relief over a 12-month period.

## Methods

### Patient characteristics and inclusion criteria

Between December 2012 and April 2014, 125 patients with symptoms of pelvic pain, heavy menstrual bleeding and/or dysmenorrhea were screened with MRI and were diagnosed with adenomyosis uterus. Seventy-three of the patients screened were candidates for the MRgFUS procedure. Thirty-seven of the screened candidates without fibroids, with symptoms of heavy menstrual bleeding and dysmenorrhoea attributed to MRI-suspected adenomyosis and who wished to preserve their uterus were eligible for this study.

The MRI diagnosis of adenomyosis was defined as junctional zone thickness of >12 mm. A diagnosis of focal adenomyosis was made when there was hypertrophic, distorted and localized low-signal-intensity region within the myometrium and a diagnosis of diffuse adenomyosis was made when diffuse thickening of the endometrial-myometrial junctional zone along with homogeneous low signal intensity was found scattered throughout the uterine musculature, on T2-weighted MRI sequences. Those that did not fall into this criterion or had abdominal wall thickness measuring over 3.5 cm were not candidates for MRgFUS treatment. Patients were excluded from the analysis if they had coexisting endometriotic cysts diagnosed on MRI, were treated with GnRH agonist or an intrauterine device after MRgFUS treatment.

Thirty-seven patients (31.2 %) who fell in the study inclusion criteria opted for and were treated by MRgFUS. Only 26 patients came back for follow-up at 3, 6 and 12 months and were included in the analysis. Eleven patients (28.2 %) did not return for the 12-month follow-up. Of the 26 patients (66.7 %), two patients required a second sitting due to diffuse adenomyosis, as regions were treated separately. The scoring for these patients was done from the second sitting which was done at 1 month interval from the first sitting. Of the 26 patients included in the analysis, two patients have been followed for 24 months.

All patients were counselled on the nature of the procedure and all provided informed consent for this form of treatment. The institutional review board of Womens Center, Coimbatore, India approved this retrospective cohort study.

SSS focussing on heavy menstrual bleeding and dysmenorrhea was assessed through a questionnaire from all patients included in the study, collected on the day of treatment and up to 12 months (26 patients) and 24 months (2/26 patients) following treatment.

### MRgFUS procedure

Patients were put on a liquid diet a day prior to the procedure. On the day of the procedure the bladder was catheterized and the patients were given enema for bowel preparation. Rectal filling with ultrasound gel was done for all patients prior to the starting the procedure in order to push the uterus anteriorly and prevent movement during sonications. Mild conscious sedation with fentanyl IV was administered prior to the start of the treatment with subsequent sedation administered during treatment to retain patient comfort and reduce movement during the procedure. Patients were treated in the prone position. The region of treatment (ROT) was planned as multiple sonication spots measuring 3 to 5 mm. The therapeutic ultrasound beam was delivered to a target area of adenomyosis raising the local temperature to 70–90° Celsius in a matter of seconds producing thermal coagulation of the sonicated spot. The treated area was mapped on the software and the operator repeatedly sonicated spots until the planned area of adenomyosis was ablated. Safety was assured with fiducials drawn around the ROT indicating patient movement, real time monitoring performed with magnitude images and temperature feedback. Post-treatment contrast imaging with IV gadolinum was performed to clearly delineate the treated area (s). Only one region was treated per session. Therefore, patients with focal adenomyosis were treated in a single sitting and patients with diffuse adenomyosis were treated in 2 sittings. The average time duration per sitting was 4 h.

### Symptom scoring

Due to the absence of an adenomyosis-specific questionnaire, a questionnaire designed to assess fibroid symptoms was used. A score from 8 questions (UF-QOL) on the SSS was transformed to a 0–100 score [18]. A separate scoring on a scale of 1 to 5 was done for dysmenorrhea, 1 being lowest and 5 the highest, as the (UF-QOL) does not include it (see Additional file 1).

### Statistical analysis

Data was analysed utilising GraphPad Prism version 6.0 f. All data were subject to and passed the D’Agostino & Pearson omnibus normality test. One-way Anova analysis (SSS, dysmenorrhea and heavy menstrual bleeding) was performed to determine statistical significances and a *p*-value of <0.05 was considered as significant. Pearson’s correlation coefficient analysis was performed to determine *r*-value for correlation between heavy menstrual bleeding versus dysmenorrhea over time and a 95 % confidence band of the best-fit line was utilised.

## Results

### Patient demographics

The median age of the patients was 33.5 (23–49) with a median BMI of 26 (19–31.5; Table 1). Of the 26 patients with follow-up at 12 months, 24 were treated for focal adenomyosis (involving either the anterior wall, posterior wall, or fundus) and 2 were treated for diffuse global adenomyosis (entire uterus enlarged and globular).

**Table 1:** Patient demographics

Total no: of patients	26
Median age (age range)	33.5 (23–49)
Median BMI (BMI range)	26 (19–31.5)
Focal Adenomyosis	24
Diffuse Adenomyosis	2

### Symptom outcomes

**Fig. 1:**
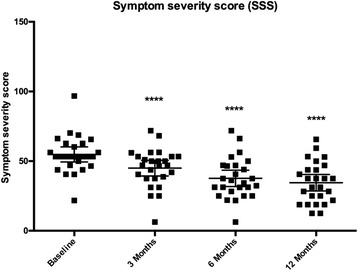
Reduction of symptom severity score (SSS) in patients undergoing MRgFUS. Scatter dot plot of the mean SSS in 26 adenomyosis patients undergoing MRgFUS treatment at baseline, 3, 6 and 12 months. One-way ANOVA analysis indicated a significant reduction in the mean score at 3, 6 and 12 months compared to baseline SSS (**** - *p* < 0.0001)

The 26 patients were included for data analysis and the SSS score for these patients at baseline, 3, 6 and 12 months was calculated based on the UF-QOL score. The mean SSS for each of these time points indicated was significantly reduced to 44.95 ± 2.72, 37.64 ± 2.85 and 34.50 ± 2.89 (*p* < 0.0001) at 3, 6 and 12 months respectively compared to mean SSS of the baseline (54.83 ± 2.63) (Fig. 1). This significant reduction indicates a likely improvement in the quality of life for these patients. Of the 26 patients, two patients had follow-up data at 18 and 24 months and in both of these patients there was a slight increase in SSS from the 12-month time-point.

**Fig. 2:**
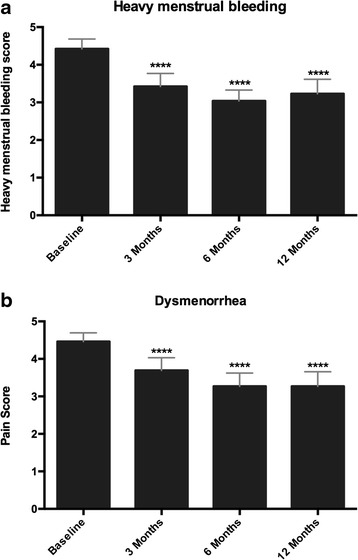
**a** Dysmenorrhea significantly reduces over time. Bar graph of the mean dysmenorrhea score at baseline, 3, 6 and 12 months in 26 adenomyosis patients undergoing MRgFUS treatment. One-way ANOVA analysis indicated a significant reduction in the mean score at 3, 6 and 12 months compared to baseline dysmenorrhea score (**** - *p* < 0.0001). **b** Heavy menstrual bleeding significantly reduces over time. Bar graph of the mean heavy menstrual bleeding score in 26 adenomyosis patients undergoing MRgFUS treatment at baseline, 3, 6 and 12 months. One-way ANOVA analysis indicated a significant reduction in the mean score at 3, 6 and 12 months compared to baseline menorrhagia score (**** - *p* < 0.0001)

Heavy menstrual bleeding and dysmenorrhea scores are one of the eight components that formed part of the SSS (UF-QOL). Mean baseline heavy menstrual bleeding score (4.42 ± 0.12) compared to the 3, 6 and 12-month time-points (3.42 ± 0.16, 3.03 ± 0.14 and 3.23 ± 0.18) was found to significantly improve over time in MRgFUS patients (*p* < 0.0001, Fig. 2a). Likewise, mean baseline (4.46 ± 0.11) dysmenorrhea score compared to 3, 6 and 12- month score (3.69 ± 0.16,3.26 ± 0.17 and 3.26 ± 0.18) was found to be significantly reduced (*p* < 0.0001, Fig. 2b).

**Fig. 3:**
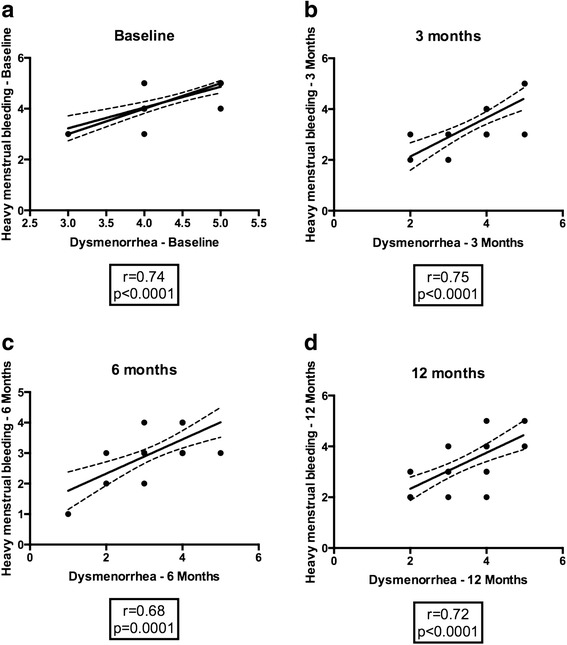
**a**-**d** Correlation of dysmenorrhea vs heavy menstrual bleeding at baseline, 3, 6 and 12 months. Correlation plot of dysmenorrhea vs menorrhagia at **a** baseline, **b** 3 months, **c** 6 months and **d** 12 months indicates a significant correlation across all time points with *r* and *p* values as indicated below each graph

Given that both dysmenorrhea and heavy menstrual bleeding were significantly reduced over time in our MRgFUS patients (Fig. 2a, b), the scores at matched time-points were interrogated for correlation. Heavy menstrual bleeding positively correlated with dysmenorrhea in MRgFUS patients. Analysis revealed that was a strong positive correlation (baseline, *r* = 0.74; 3 month, *r* = 0.75; 6 month, *r* = 0.68; 12 month, *r* = 0.72; *p* < 0.0001) at all time points between heavy menstrual bleeding and dysmenorrhea in these patients (Fig. 3a-d).

### Uterine imaging

A review MRI was suggested for patients at 6 and 12 months to assess the reduction in adenomyotic volume. Out of the 26 patients, only 3 patients consented to have an MRI review at both 6 and 12 months so uterine volume assessment over time could not be calculated for the entire study group. Of the three patients, all three had reduction of adenomyotic volume at 6 months (2, 54, 37 %), which reduced further in one patient and a slight increase was seen in two (9, 44, 32 %) with no changes in SSS.

### Fertility outcome

Of the 26 patients, 13 patients were infertile and planned to start treatment for the same after the procedure. The median age of those seeking fertility treatment was 34. Of the 26, one patient who was not seeking treatment for fertility conceived spontaneously 4 months post MRgFUS. Of those seeking fertility treatment, only five patients underwent assisted reproductive technology (ART) due to cost constraints, of which two patients conceived at 12 and 18 months post MRgFUS respectively. Both patients delivered at term, one vaginally and the other delivered through an emergency caesarean section.

## Discussion

Advances in treatment for adenomyosis have been limited by the difficulties in determining a clinical diagnosis, appropriately defining the extent of the lesion and the lack of a specific intervention. Different surgical and medical modalities of treatment have been addressed in the literature but many of these have not been tested specifically for adenomyosis uteri. In the absence of treatments directed at the disease itself, management is often directed at the symptoms [19]. MRgFUS has been used effectively for the non-invasive ablation of pathologic tissue [9, 14]. It has been hypothesized that MRgFUS could also treat adenomyosis accurately and successfully without deleterious effects on the surrounding myometrium and on subsequent fertility [15].

In our study, we utilised MRgFUS for adenomysis patients and found that there was significant reduction of SSS at 3, 6 and 12 months post-treatment (44.95 ± 2.72, 37.64 ± 2.85, 34.50 ± 2.89 respectively). Of the 26 patients, two patients had follow-up data at 18 and 24 months and, in both of these patients there was a slight increase in SSS at 18 and 24 months (Fig. 1b). This finding is in line with the nature of the disease and the possibility of recurrence. In addition, none of the patients included in our analysis received hormonal injections or any other interventions post MRgFUS.

Women with diffuse adenomyosis are often not considered as candidates for conservative surgery [20]. In our study, of the 37 patients who were treated by MRgFUS, a total of 6 (16.21 %) patients had diffuse adenomyosis. But of this number only 2 were included in the analysis as the remaining patients were lost to follow up. Of the two patients for whom we had follow up at 3,6 and 12 months, one patient had significant improvement of SSS from a baseline score of 66.25 % (50.0, 50.0 and 46.87 % respectively). The other patient had reduction of SSS at 3 months (25.0 %) and 6 months (25.0 %) from a baseline score of 40.62 %. But her score increased to 65.60 % by 1 year post treatment. Further studies with larger number of patients are required to support the use of MRgFUS in treating diffuse adenomyosis effectively.

Amongst women with symptomatic adenomyosis, heavy menstrual bleeding and dysmenorrhea are the two major grievances [21]. Several publications describe short term and long term follow-up of SSS in patients who have been treated using MRgFUS for adenomyosis indicating that the treatment was safe and that there was significant reduction in symptoms. In all these studies, the assessments of SSS have been based on the UF-QOL questionnaire. None of these papers specifically look at dysmenorrhea, the most disabling symptom [11, 16, 17]. There is also a lack of consensus in the literature regarding the relationship between adenomyosis and heavy menstrual bleeding [22].

In our study, we assessed dysmenorrhea separately as it is not a part of the UF-QOL questionnaire. We found a significant reduction in dysmenorrhea at 3, 6 and 12 months respectively (Fig. 2a). In the two patients for whom we had an 18 and 24 month follow-up, we found that there was sustained relief of dysmenorrhea up to 24 months. In addition, when heavy menstrual bleeding was assessed individually, there was a significant reduction in the scoring at 3, 6 and 12 months respectively (Fig. 2b). Heavy menstrual bleeding and dysmenorrhea are both positively associated with the depth of penetration of individual adenomyotic glands into the myometrium [1, 23]. In our study we found a positive correlation between the reduction of heavy menstrual bleeding and dysmenorrhea at 3, 6 and 12 months.

## Limitations of the study

The first limitation of the study was the absence of a tool for evaluating patients with the spectrum of symptoms related specifically to adenomyosis, we had to evaluate symptoms of patients before and after treatment of adenomyosis using MRgFUS with the help of a questionnaire which is traditionally used to evaluate symptoms associated with fibroids. We had to design a score specifically for assessing dysmenorrhoea, as it was not a part of the UF-QOL questionnaire. The next limitation was the difficulties faced to get follow-up at 3, 6 and 12 months for all treated patients thereby limiting our study population. Patients who could not come in to the hospital for assessment were followed up through phone calls. Some patients were lost to follow up after a single post treatment assessment. In order to evaluate the overall size reduction in the extent of adenomyosis and the overall uterine volume post MRgFUS treatment, an MRI scan was suggested at 6 and 12 months follow up visit. But as a majority of Indian insurance companies did not cover the procedure during the study period; MRI assessment at both 6 and 12 months follow-up was performed only for 3/26 patients. This was a major draw back faced during the study as the symptomatic changes could not be correlated with the structural changes in the uterus post treatment in significant number of patients. Of the three patients with MRI follow-up, adenomyotic volume reduced at 6 months, which reduced further in one patient and a slight increase was seen in the other two. Despite the increase in volume, there was no change in the SSS. This is significant as it also shows that increase in volume may not necessarily cause an increase in the symptoms of dysmenorrhea and heavy menstrual bleeding.

The role of adenomyosis in infertility is still debated: the association between adenomyosis and sterility is poorly understood (occurring in between 1 and 14 % of the cases in the literature) but the frequency of infertility has not been assessed in large studies [24]. There is no consensus on appropriate management of symptomatic adenomyosis in women seeking fertility [25]. The concept of conservative uterus – sparing surgery is increasing but it has not yet become the standard [26]. It involves techniques that modify the anatomy of the uterus and this may contribute to a declined post pregnancy rate [27]. Of those seeking fertility treatment post MRgFUS in our study population (13/26), treatment for infertility was started 3 months post MRgFUS. Two patients conceived with assisted reproductive technology (ART) with ongoing pregnancies at 12 and 18 months post MRgFUS respectively. Their infertility workup did not demonstrate any abnormal findings other than an enlarged uterus. Of the 26, one patient who was not seeking treatment for fertility conceived spontaneously 5 months post MRgFUS. Her pregnancy was uneventful and she gave birth to a healthy male baby at term following a normal vaginal delivery.

## Conclusion

In summary, this study is significant as it provides evidence that for symptomatic adenomyosis, focal or diffuse, MRgFUS can be used as a safe and effective treatment modality for immediate as well as sustained improvement in quality of life, especially in respect to the most significant symptoms of heavy menstrual bleeding and dysmenorrhoea, without any add back treatment such as GNRH analogues or Levonorgestrel IUD (LNG). Since MRgFUS is a non-invasive treatment modality, it should be feasible to perform MRgFUS for patients even with relapsed adenomyosis. In our center, we have on going studies on the time interval to relapse of adenomyosis following MRgFUS and effectiveness of combined approach of MRgFUS followed by LNG insertion for sustained symptomatic relief in patients as an alternate to hysterectomy. Further larger studies are needed to refine the optimal patient population for this procedure and to assess how the MRgFUS compares to other uterus sparing treatment modalities.

## Additional file

Additional file 1: UF-QOL SSS. (PDF 171 kb)
