# Supplementary material for: Heavy menstrual bleeding and dysmenorrhea are improved by Magnetic Resonance Guided Focused Ultrasound Surgery (MRgFUS) of adenomyosis
Source: Fertil Res Pract. 2016 May 16;2:8. doi: 10.1186/s40738-016-0021-x (PMC5424318; doi:10.1186/s40738-016-0021-x)
Supplement: Additional file 1: — UF-QOL SSS. (PDF 171 kb) [file 40738_2016_21_MOESM1_ESM.pdf]

Pt. Initials: ________ Pt. ID: ____________

Date: ____________

UTERINE FIBROID SYMPTOM AND HEALTH-RELATED QUALITY OF LIFE QUESTIONNAIRE (UFS-QOL)

Listed below are symptoms experienced by women who have uterine fibroids. Please consider each symptom as it relates to your uterine fibroids or menstrual cycle. Each question asks how much distress you have experienced from each symptom during the previous 3 months.

There are no right or wrong answers. Please be sure to answer every question by checking (✓) the most appropriate box. If a question does not apply to you, please mark "not at all" as a response.

During the previous 3 months, how distressed were you by...	Not at all	A little bit	Some-what	A great deal	A very great deal
1. Heavy bleeding during your menstrual period	☐ 1	☐ 2	☐ 3	☐ 4	☐ 5
2. Passing blood clots during your menstrual period	☐ 1	☐ 2	☐ 3	☐ 4	☐ 5
3. Fluctuation in the duration of your menstrual period compared to your previous cycle	☐ 1	☐ 2	☐ 3	☐ 4	☐ 5
4. Fluctuation in the length of your monthly cycle compared to your previous cycles	☐ 1	☐ 2	☐ 3	☐ 4	☐ 5
5. Feeling tightness or pressure in your pelvic area	☐ 1	☐ 2	☐ 3	☐ 4	☐ 5
6. Frequent urination during the daytime hours	☐ 1	☐ 2	☐ 3	☐ 4	☐ 5
7. Frequent nighttime urination	☐ 1	☐ 2	☐ 3	☐ 4	☐ 5
8. Feeling fatigued	☐ 1	☐ 2	☐ 3	☐ 4	☐ 5
